# Securing the replication ground: how barley stripe mosaic virus safeguards the host chloroplast envelope

**DOI:** 10.1093/plcell/koag195

**Published:** 2026-06-25

**Authors:** Ved Prakash

**Affiliations:** Assistant Features Editor, The Plant Cell, American Society of Plant Biologists; Department of Plant Pathology, College of Food, Agriculture, and Environmental Sciences, The Ohio State University, 1680 Madison Ave, Wooster, OH 44691, United States

Chloroplasts function as a metabolic hub, generating energy rich molecules (ATP and NADPH) for fixing CO_2_ into sugar needed for plant growth. In addition, chloroplasts also function as a vital command center for plant development and stress mitigation (Jarvis and López-Juez 2013). Because of this crucial role, invading pathogens frequently target chloroplasts to suppress immunity. In particular, viruses either suppress chloroplast-mediated immunity (Xiao et al. 2025) or physically disrupt the organelle during replication (Bhattacharyya et al. 2015). Plants employ quality control systems such as the Ubiquitin-Proteasome System (UPS) and autophagy to degrade stress-damaged chloroplasts. While research has increasingly mapped out how plants use quality control systems to maintain the proper functioning of chloroplasts, our understanding of how viruses navigate and dynamically manipulate these organelle destruction pathways to ensure their own survival has remained fragmented.

In this issue, Yanlin Chen and colleagues (Chen et al. 2026) uncover a molecular plant-viral tug-of-war at the chloroplast outer envelope. They report a tripartite regulatory network where host defense and viral adaptation meet for the integrity and stability of chloroplast translocon protein Toc34. The finding centers on pheophytinase (PPH), a chloroplast outer envelope-localized hydrolase, traditionally recognized for its function in chlorophyll degradation during leaf senescence, abiotic stress, and darkness (Guyer et al. 2014; Zhang et al. 2016; Teng et al. 2021). The authors reveal that PPH plays a crucial role in broad-spectrum antiviral defense. Under viral infection, PPH acts as a molecular scaffold at the chloroplast surface by physically interacting with Toc34, which is required for protein import and membrane integrity. PPH recruits the cytosolic E3 ubiquitin ligase PUB4 to Toc34, leading to its polyubiquitination and destruction by the UPS. Degradation of Toc34 disrupts the chloroplast membrane structure, which is required for viral replication. As such, the PPH-PUB4-Toc34 module creates an inhospitable cellular environment that restricts viral proliferation (see Fig. 1).

**Figure 1:**
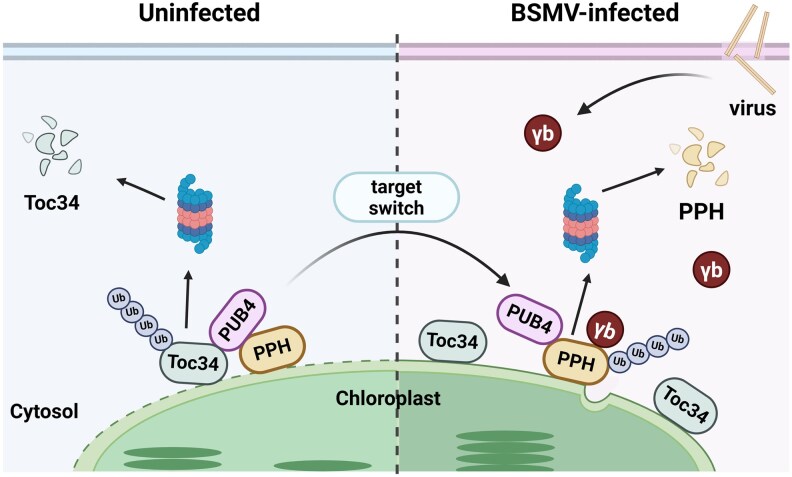
A proposed model illustrating the pheophytinase (PPH) mediated ubiquitination and degradation of chloroplast translocon proteins Toc34 via E3 ubiquitin ligase PUB4 to restrict virus infection (left). However, γb protein of barley stripe mosaic virus (BSMV) physically interacts with chloroplast membrane and PPH. This interaction pushes PUB4 away from Toc34 and redirects PUB4 toward PPH for its degradation, maintaining membrane integrity for virus replication (right). Adapted from Chen et al., (2026), Fig. 7.

However, the co-evolving pathogen barley stripe mosaic virus (BSMV) employs a mechanism which overturns the PPH-PUB4 mediated degradation of Toc34 (Chen et al. 2026). BSMV relies on chloroplast membranes for anchoring its viral replication complexes. To prevent the PPH-driven destruction of its replication ground, BSMV uses a multifunctional effector protein γb, which physically interacts with the chloroplast envelope by directly binding to PPH. Interestingly, γb flips the cellular wiring of the scaffold complex PPH: γb pushes PUB4 away from the critical Toc34 translocon and redirects the E3 ligase dependent ubiquitination toward PPH itself (Chen et al. 2026) (see Fig. 1). As such, it functions as a biochemical redirector that leads to the rapid proteasomal degradation of PPH, stabilizing Toc34 levels and saving the chloroplast envelope structure from destruction. By degrading PPH, the virus preserves the functional state of the chloroplast membrane required for its multiplication, linking chloroplast homeostasis with viral fitness.

By identifying PUB4 as a bidirectional molecular switch, the authors show how BSMV has adapted to exploit the host's own post-translational machinery for their benefit. This finding repositions a well-known metabolic enzyme (PPH) as a regulator of cellular immunity. Importantly, the uncovering of the PPH-PUB4 regulatory pathway provides plant scientists with an innovative genetic blueprint for engineering broad-spectrum resistance against destructive plant viruses.

## Recent related articles in *The Plant Cell*

- Wang et al., (2026) demonstrated that p23 protein of beet black scorch virus (BBSV) induces NbSec62-mediated ER-phagy to regulate the balance between viral replication and ER homeostasis in *Nicotiana benthamiana*. They also show NbSec62-mediated ER-phagy during tobacco mosaic virus and turnip mosaic virus infection.
- Zuo et al., (2025) reported that glyceraldehyde 3-phosphate (GAP), one of the metabolites translocated by triose phosphate/phosphate translocator (TPT) in the inner membrane of the chloroplast envelope, enhances the expression of defense-related genes and induces signaling pathways to restrict viral infection in *Arabidopsis*.
- Luan et al., (2025) reported that the *Arabidopsis* dual-specificity phosphatase DSP4 is a negative regulator of plant immunity against turnip mosaic virus (TuMV). They identified a molecular mechanism by which TuMV P3 exploits membrane-associated phosphatase to promote virus infection.

## Data Availability

No new data were generated or analysed in support of this research.

## References

[koag195-B1] Bhattacharyya D et al 2015. A geminivirus betasatellite damages the structural and functional integrity of chloroplasts leading to symptom formation and inhibition of photosynthesis. J Exp Bot. 66:5881–5895. 10.1093/jxb/erv299. 26113193 PMC4566980

[koag195-B2] Chen Y et al 2026. A viral protein-driven ubiquitination switch fine-tunes chloroplast outer envelope proteins homeostasis. Plant Cell. 38:koag179. 10.1093/plcell/koag179. 42276832

[koag195-B3] Guyer L et al 2014. Different mechanisms are responsible for chlorophyll dephytylation during fruit ripening and leaf senescence in tomato. Plant Physiol. 166:44–56. 10.1104/pp.114.239541. 25033826 PMC4149727

[koag195-B4] Jarvis P, López-Juez E. 2013. Biogenesis and homeostasis of chloroplasts and other plastids. Nat Rev Mol Cell Biol. 14:787–802. 10.1038/nrm3702. 24263360

[koag195-B5] Luan Y et al 2025. A plant RNA virus hijacks a dual-specificity phosphatase to attenuate MAPK-mediated immunity for robust infection. Plant Cell. 37:koaf232. 10.1093/plcell/koaf232. 40990618

[koag195-B6] Teng K et al 2021. Functional characterization of the pheophytinase gene, ZjPPH, from Zoysia japonica in regulating chlorophyll degradation and photosynthesis. Front Plant Sci. 12:786570. 10.3389/fpls.2021.786570. 35003174 PMC8733386

[koag195-B7] Wang R et al 2026. Sec62 restricts ER-replicating positive-strand RNA virus infections via UPR-dependent ER-phagy. Plant Cell. 38:koag014. 10.1093/plcell/koag014. 41592053

[koag195-B8] Xiao K et al 2025. A fungal effector promotes infection via stabilizing a negative regulatory factor of chloroplast immunity. Nat Commun. 16:6970. 10.1038/s41467-025-62326-4. 40730546 PMC12307619

[koag195-B9] Zhang J et al 2016. Functional characterization and hormonal regulation of the PHEOPHYTINASE gene LpPPH controlling leaf senescence in perennial ryegrass. J Exp Bot. 67:935–945. 10.1093/jxb/erv509. 26643195 PMC4737083

[koag195-B10] Zuo DP et al 2025. A triose phosphate/phosphate translocator triggers antimicrobial immunity by exporting glyceraldehyde 3-phosphate from chloroplasts. Plant Cell. 37:koaf245. 10.1093/plcell/koaf245. 41092103

